# Development of Competitive ELISAs Suitable for Detection of Recombinant Human Growth Hormone in Illegal Nutritional Supplements

**DOI:** 10.1155/jamc/3021570

**Published:** 2025-06-06

**Authors:** L. Karamonová, B. Procházková, M. Volný, A. Nemeškalová, D. Sýkora, M. Kuchař, L. Fukal, B. Holubová

**Affiliations:** ^1^Department of Biochemistry and Microbiology, University of Chemistry and Technology Prague, Prague, Czech Republic; ^2^Department of Analytical Chemistry, University of Chemistry and Technology Prague, Prague, Czech Republic; ^3^Forensic Laboratory of Biologically Active Substances, Department of Chemistry of Natural Compounds, University of Chemistry and Technology Prague, Prague, Czech Republic; ^4^Psychedelics Research Centre, National Institute of Mental Health, Klecany, Czech Republic

**Keywords:** black market, competitive ELISA, doping, growth hormone, nutritional supplements

## Abstract

The use of human growth hormone (hGH) by athletes remains widespread despite being banned by the World Anti-Doping Agency (WADA). Current commercial ELISA kits for the determination of hGH are predominantly based on the sandwich format of this method, which allows the analysis of the whole hGH molecules, but not their fragments. The abuse of these fragments by athletes is the current trend in doping. Therefore, a competitive ELISA format is needed; however, only a limited number of kits on the market are based on this principle. Furthermore, these kits are designed to detect and quantify hGH only in blood plasma, serum or tissue cultures. When analysing a nutritional supplement containing hGH using one of these ELISA kits, a false negative result was observed. In the present work, two competitive ELISA methods for the detection of hGH in illegal nutritional supplements were developed. The development of the methods involved the selection of suitable immunoreagents and their combinations with subsequent optimisation of the conditions for their use. The methods were then characterised (with LOD 0.4 and 18.4 ng/mL, according to the primary antibody) and applied to analyse real nutritional supplements. In all 34 samples suspected of containing hGH, the hormone was detected using both ELISA methods and subsequently confirmed by LC-UV and LC-MS. Therefore, the newly developed ELISA methods could become a valuable tool for the police in identifying illegal preparations.

## 1. Introduction

Growth hormone (GH), also known as somatotropin, is a polypeptide hormone that has anabolic and growth-promoting properties. Although there are several molecular variants (isoforms) of human growth hormone (hGH), the monomeric 22 kDa-GH, a single-chain protein of 191 amino acids with two disulphide bridges, is the most common form of hGH present in the pituitary gland and blood. Using recombinant technologies, this isoform is also produced commercially for medical purposes and is referred to as ‘recombinant hGH (rhGH)'. It is also a very widespread type of doping [1–4]. Abuse of hGH in sports is popular due to its alleged anabolic capabilities, lipolytic activities and difficulties in detection. hGH selectively boosts anaerobic sprint capacity in athletes; however, it has not been shown to significantly increase muscle strength, power output or maximum oxygen consumption [5–7]. Long-term use of high doses of hGH, on the other hand, is associated with major health risks that are often underestimated by consumers [1, 3]. Since 1989, when it became clear that the advent of biotechnology products based on DNA recombination would make hGH far much more available on the regular and black markets, international federations and the International Olympic Committee have included hGH on the list of prohibited chemicals [8]. The whole hGH and its fragments are listed on WADA's Prohibited List under the category of Peptide Hormones, Growth Factors, Related Substances and Mimetics (S2). The 2025 WADA Prohibited List categorises performance-enhancing substances under several headings, including ‘Prohibited at all times', ‘Prohibited in competition' and ‘Prohibited in particular sports'. The classification of category S2 as ‘Prohibited at all times' indicates that its use is forbidden both during and outside of competition [1, 9, 10]. Despite these restrictions, GH and its derivatives are still abused. A 2007 inquiry into the use of performance-enhancing drugs in Major League Baseball as well as the infamous Biogenesis scandal of 2013 revealed that many professional baseball players were linked to hGH use, thus confirming the widespread abuse of hGH among professional athletes. An abuse of hGH is a known problem even in juvenile athletics, as up to 5% of high school athletes report its use [11].

However, doping is no longer limited solely to the abuse of full-length rhGH; recent trends also involve the use of individual fragments of its polypeptide chain. In fact, structural and functional studies have demonstrated that fragments of the hGH molecule can retain lipolytic activity while avoiding the diabetogenic effects typically associated with the complete hormone. Notably, the C-terminal peptide comprising amino acids 177–191 (also known as AOD9401), along with other hGH fragments, has recently emerged as a widely abused substance [12, 13].

In vitro bioassays, radioreceptor assays and immunoassays can all be used to detect hGH in biological fluids. While bioassays and radioreceptor tests are generally reserved for research purposes, immunoassays are suited for routine usage such as anti-doping control. Their readouts include radioactivity, colourimetry, fluorescence or chemiluminescence [5, 6, 12, 14]. Most antibodies used in immunoassays recognise all GH isoforms [15, 16], although there are a few assays specific for 22K-GH [16–18], 20K-GH [17, 19] and placental GH [20]. To identify hGH abuse, two procedures based on population-derived decision limits are now used: the hGH Biomarkers Test and the Isoforms Differential Immunoassay. The former is based on the detection of sensitive markers of rhGH biological activity, whereas the latter, the analytical method, focuses on the detection of GH molecular isoforms. Both are currently limited to blood samples [3, 5, 21–23].

Despite the efforts of anti-doping agencies to limit doping in sports, the usage of performance enhancing substances and methods remains an unsolved problem [24]. The rise of darknet, which allows users to obtain illicit substances anonymously, further deepens the problem. In 2009, a doping control laboratory in Germany tested 11 different seized goods. Only four of them contained the substances and quantities indicated on the label. Regarding hGH, two of them were labelled as containing hGH, but only one vial had hGH presence verified by LC-MS [25]. This trend was confirmed in the following years by another German study of 337 black market products, which were analysed mostly by LC-MS or GC-MS. Drugs that were relevant as performance-enhancing agents were detected in 311 samples, with peptide hormones (mainly hGH) accounting for 12.8% of the positive findings. Another important finding was that only 43% of the products on the black market contained the substances declared on the labels of the respective packaging. Such a low percentage shows that many covert underground laboratories either lack the skills and tools necessary to produce appropriate compounds with adequate quality, or they knowingly endanger the health of their customers to generate a distributor's profit [26]. A Norwegian investigation, which used LC-MS and GC-MS to examine seized material suspected of carrying doping agents, provided a similar indication of doping substances available on the darknet. The presence of peptide or protein hormones was suspected in 94 out of 296 black market items. According to the results of the examination, 20% of the suspect products did not include any illegal drugs, while hGH was found in 21% of the suspect samples [27]. McLean et al. conducted a descriptive analysis of the availability, quantity, price and forms of substances included on the WADA International Standard List of Prohibited Substances for 2021 that were for sale on the darknet markets [28]. In addition to a significant range of anabolic compounds, hormonal and metabolic modulators, diuretics and masking agents, peptide hormones such as GH have been shown to be available on the darknet. This study also confirmed that the use of black market items poses a substantial health risk, as many products did not contain the ingredients specified on the label or contained other ingredients not listed on the label.

State police and customs authorities are working to curb the trade and distribution of performance-enhancing drugs. At their request, unknown pharmaceutical products suspected of containing peptide drugs are regularly subjected to analysis [24, 29]. The method commonly used by the Police of the Czech Republic for detecting hGH in suspect products is LC-MS. However, the internal protocol requires verification of the results by an alternative analytical method. In addition, the LC-MS detection of hGH is based on comparison of the retention time and the accurate mass, which by itself does not definitively confirm the hormone's identity. To overcome this limitation, immunoassays employing specific antibodies could be used for verification and thereby serve as a suitable confirmatory method. Moreover, enzyme-linked immunosorbent assay (ELISA) is considered a fast, simple and easily accessible method that does not require sophisticated instruments. Although it has the potential for large-scale testing of confiscated samples from the darknet, no ELISA specifically designed for this type of sample has yet been published.

Commercial ELISA kits are designed for the detection of hGH only in biological samples, such as serum, plasma and cell culture supernatants. These tests are based predominantly on the sandwich format of ELISA (kits from Abcam, Sigma-Aldrich, RayBiotech, LS Bio, Anogen, Antibodies, Invitrogen, Proteintech, Bio-Techne, OriGene …). In general, sandwich ELISA has a lower detection limit than competitive ELISA, and because it uses two antibodies targeting different epitopes, it may also exhibit higher specificity. However, when high-quality antibodies are used and appropriate conditions are selected, the competitive ELISA format can also be highly specific. In the case of analysing illegal nutritional supplements, where hGH concentrations are reported in mg/mL, the requirement for low limit of detection (LOD) is not critical. A very important consideration, however, is the potential future expansion of target analytes from full-length hGH molecule to a wide range of its fragments, the abuse of which is a growing trend in doping [12, 13]. Due to their limited size, typically consisting of only one potential epitope for detecting antibody, the competitive ELISA format is the only suitable detection method. Unfortunately, there are currently only two commercially available competitive ELISA kits, both designed to detect hGH in biological materials: Human GH (Competitive EIA) ELISA Kit (LS Bio) and Human GH ELISA Kit (MyBioSource), both currently without relevant references regarding their successful use in hGH detection.

The aim of this study was to evaluate an existing commercial competitive ELISA kit, designed for the determination of hGH in biological materials, to see if it could be used for detecting hGH in illegal nutritional supplements and, if necessary, to develop a new ELISA method that could serve as a suitable tool for the Police of the Czech Republic in detecting this hormone in suspect products.

## 2. Materials and Methods

### 2.1. hGH Standards

For the expression of complex mammalian proteins, eukaryotic expression systems are typically recommended. Although their production processes are stringent, time-consuming and expensive, eukaryotic cells can perform complex post-translational modifications (such as glycosylation and phosphorylation) and ensure the proper folding of expressed proteins. Therefore, human embryonic kidney (HEK 293) cells are commonly used by companies such as Sigma-Aldrich and Proteintech Group to produce rhGH. However, because the predominant 22 kDa isoform of hGH does not undergo post-translational modifications, some producers, such as PeproTech EC, employ a simpler and less expensive bacterial expression system. Additionally, bacteria *Escherichia coli*, commonly used for protein expression, grow rapidly and at high densities, resulting in higher yields of the expressed protein. Since the expression systems used for rhGH in illicit nutritional supplements are unknown, standards prepared using both expression systems (HEK 293 and *E. coli*) were tested to obtain suitable immunoreagents for ELISA (see Table 1).

Individual stock solutions of hGH standards with a concentration of 100 μg/mL were prepared by dissolving accurately weighed amounts in deionised water; only hGH from Proteintech Group was dissolved in 1× PBS, pH 7.4, according to the manufacturer's recommendations. All solutions were aliquoted and stored at −20°C.

### 2.2. Samples

In total, 34 suspect samples were analysed, 13 of which were originally confiscated by the Police of the Czech Republic, and the remaining 21 were kindly provided by the analytical laboratory Janoshik s.r.o. Most samples were obtained in powder form in injection vials; five samples (Sample 6 and Samples 10–13) were received as an injection solution in a vial, and Sample 21 was in the form of a pen filled with injection solution. Some vials were labelled with the declared hGH content (in mg and/or IU) in addition to the product name, while others were unlabelled (see Table 2). All samples in powder form were dissolved in 1 mL of deionised water, except for Sample 5, which was dissolved in 0.5 mL of deionised water to achieve a similar concentration (3.3–3.7 mg/mL). Samples 6, 10, 11 and 13 were already in solution form with a volume of 5 mL and a declared hGH content of 50 IU (16.65 mg), which is equivalent to 3.3 mg/mL of hGH. The label on Sample 21 (HUTROPE LinePen) indicated an hGH content of 10 mg, which, given a fill volume of 3 mL, corresponds to a concentration of 3.33 mg/mL.

The prepared solutions of most samples were colourless and clear after dissolution. However, some samples exhibited turbidity (Samples 16, 19, 25, 28 and 34), and in some cases, a larger amount of insoluble impurities was observed (Samples 17, 26 and 27). Sample 32 was clear after dissolution but exhibited a distinct yellow colouration. This indicates that the matrices of the individual samples are considerably different.

### 2.3. Competitive ELISA Commercial Kit

Commercial Human GH ELISA Kit (Catalogue Number MBS284646, MyBioSource Inc., San Diego, CA, USA) was used for quantitative detection of hGH in Sample 1 confiscated by the Police of the Czech Republic and also in model samples spiked by hGH standards from all three producers: Sigma-Aldrich, Proteintech Group and PeproTech EC. Antibody-coated 96-well microtiter plate, biotin-conjugated hGH, streptavidin-conjugated horseradish peroxidase (HRP), HRP substrate in solution and stop solution were included in the kit. Standards labelled as S5-S0 were also provided, with declared hGH concentration of 50, 25, 12.5, 6.25, 3.12 and 0 ng/mL, respectively. The kit components were kept at 4°C until analysis, and then all samples, reagents and the microtiter plate were equilibrated at laboratory temperature. The assay was carried out in accordance with the manufacturer's instructions. 50 μL of diluted samples or standards was added to the coated microtiter plate, followed by 50 μL of biotin-conjugated hGH, which was incubated at 37°C for 1 h. The plate was washed three times with diluted wash buffer (250 μL), manually inverted and slapped dry to remove remaining liquid. The plate was then incubated at 37°C for 30 min with 50 μL streptavidin-conjugated HRP. The microplate was washed five times under the above conditions. 50 μL of substrate A and 50 μL of substrate B were added, mixed and, after 15-min incubation at 37°C, 50 μL of stop solution was applied. The absorbance (450 nm) was immediately recorded with a microplate reader (uQuant BIO-TEK, Inc., Winooski, VT, USA).

Since the kit does not include a sample diluent (the manufacturer recommends applying undiluted serum or plasma), it was necessary to identify a suitable dilution solution. This was particularly important because the declared concentration of the confiscated products is approximately 3–4 mg/mL, while the detection range of the kit is only 3.12–50 ng/mL. As a potential diluent, the S0 standard solution (containing 0 ng/mL of hGH) was tested. However, due to its limited volume, which would not be sufficient for diluting a larger number of real samples, commonly used ELISA buffers, namely, PBST and PBST-3%BSA, were also evaluated.

For the preparation of model samples spiked with hGH from the internal source (i.e., the hGH provided within the kit), two groups of samples, each with a concentration of 12.5 ng/mL, were prepared. One group was derived from the standard solution S4 (containing hGH at 25 ng/mL), while the other was derived from the standard solution S5 (containing hGH at 50 ng/mL). Both groups were diluted using S0, PBST and PBST-3% BSA. For the preparation of model samples spiked with hGH from external sources (Sigma-Aldrich, Proteintech Group and PeproTech EC), the stock solutions were diluted in S0, PBST and PBST-3% BSA to final concentrations ranging from 12.5 to 1000 ng/mL. Sample 1 was diluted 10–82,500 times, again using all three diluents: S0, PBST and PBST-3% BSA.

### 2.4. Newly Developed ELISAs

#### 2.4.1. hGH Antibodies

Several anti-hGH antibodies (Table 3) were tested. They were of both monoclonal and polyclonal type and targeted either unspecified epitopes of the hGH molecule or a specific epitope between amino acids 151–189. Monoclonal antibodies were stored at 4°C and the polyclonal at −20°C.

#### 2.4.2. Secondary Antibody Conjugated With Peroxidase

Goat-derived peroxidase-conjugated antibodies against mouse IgG (whole molecule; Product No. A4416, RRID: AB_258167) and rabbit IgG (whole molecule; Product No. A6154, RRID: AB_258284) were purchased from Sigma-Aldrich (St. Louis, MO, USA). Both conjugates were aliquoted and stored at −20°C.

#### 2.4.3. Reagents and Materials

Bovine serum albumin (BSA), Tween 20, sulphuric acid, 3,3′,5,5′-tetramethylbenzidine (TMB) and hydrogen peroxide (30%) were purchased from Sigma-Aldrich (Saint Louis, MO, USA). A set of 96-well polystyrene microtiter plates (Costar 9018) was obtained from Corning Inc. (New York, NY, USA).

#### 2.4.4. Selection of Appropriate Immunoreagents for ELISA Development

To determine appropriate immunoreagents, a direct binding of selected antibodies to immobilised standards from various producers was tested. The combinations of every hGH standard listed in Table 1 with all anti-hGH antibodies from Table 3 were sequentially examined. The following steps were taken with all possible combinations of immunoreagents: a microtiter plate (100 μL/well) was coated with a solution of hGH in 0.05M carbonate/bicarbonate buffer (pH 9.6) at concentrations ranging from 1000 to 0 ng/mL. The plate was covered and incubated overnight at 4°C. Unbound hGH was eliminated by washing (3 times, 200 μL/well) with PBST buffer (1× PBS pH 7.4 with 0.05% Tween 20 (v/v)). The microtiter plate was then filled with hGH antibody, which had been diluted in PBST-1% BSA (w/v) (100 μL/well) from 1 μg/mL with a dilution factor of 2, and the plate was incubated for 2 h at 37°C. The plate was washed as described above. Peroxidase-labelled secondary antibody was diluted 1:2500 in PBST-1% BSA and applied at 100 μL/well. Following a 1-hour incubation period at 37°C, the unbound antibody was removed as previously mentioned. Freshly prepared substrate solution (9 mL of 0.05M citrate/phosphate buffer, pH 5.0, 1 mL of TMB in DMSO (1 mg/mL) and 2 μL of 30% H_2_O_2_ (v/v)) was added (100 μL/well) and incubated for 15 min at laboratory temperature. The enzyme reaction was stopped by adding 0.5M H_2_SO_4_ (50 μL/well) and the absorbance was measured using a spectrophotometer at 450 nm (uQuant BIO-TEK, Inc., Winooski, VT, USA).

#### 2.4.5. Indirect Competitive ELISA

The indirect competitive ELISA was performed with buffers and reagents described in Section 2.4.4. Microtiter plates were coated at 4°C overnight with hGH (PeproTech EC, Cranbury, NJ, USA) in 0.05M carbonate/bicarbonate buffer (pH 9.6). Plates were then washed (3 times, 200 μL/well) with wash buffer (PBST) and immediately used or allowed to dry at laboratory temperature and stored at −20°C for later use. For the competitive ELISA step, the standards (concentration 0.1–10,000 ng/mL) or samples both diluted in assay buffer (PBST-1% BSA (w/v)) were applied to microtiter plates (50 μL/well). Antibodies were also diluted in the same buffer and added in a volume of 50 μL/well. After incubation (1.5 h, 37°C), the microtiter plates were washed (3 times, 200 μL/well) and a peroxidase-labelled secondary antibody, diluted with assay buffer, was added (100 μL/well). The plates were incubated at laboratory temperature for 1 h, and then washed again (3 times, 200 μL/well). To detect peroxidase activity, 100 μL/well of a substrate solution was added to the plates and incubated for 15 min at laboratory temperature. The enzyme reaction was stopped by adding 0.5M H_2_SO_4_ (50 μL/well) and the absorbance was measured at 450 nm.

#### 2.4.6. Interpretation of ELISA Results

Sigmoid calibration standard curves were obtained by plotting the mean values of absorbance against the logarithm of hGH concentrations through a four-parameter logistic equation. LOD was defined as the concentration of an analyte corresponding to the maximum assay signal minus 3× standard deviation (SD) in accordance with the calibration curve (the blank was calculated at least from 18 parallel determinations with the absence of an analyte). The linear working range corresponded to the analyte concentration causing the 20%–80% inhibition of the maximal assay signal. The I_50_ corresponded to the concentration of analyte giving 50% inhibition of the asymptotic maximum.

To evaluate the intra-assay variability, three separate samples were each assayed in 18 replicates on a single microtiter plate. For each sample, the coefficient of variation (CV) was calculated by dividing SD of the replicate measurements by their mean and then multiplying the result by 100 to express it as a percentage.

For inter-assay variability, the same three individual samples were analysed in triplicate on 10 separate microtiter plates. The CV for each sample was determined across the 10 plates by dividing SD of the measurements by the mean value and multiplying by 100, thus expressing the result as a percentage.

### 2.5. Instrumental Confirmation and Quantification

Sample 1 was chosen as a representative for detailed identification using high-resolution mass spectrometry. The sample was dissolved in a 1:1 (v/v) mixture of LC-MS grade acetonitrile (Sigma-Aldrich, Saint Louis, MO, USA) and water and directly introduced into the Bruker Impact II QTOF mass spectrometer (Bruker, Germany) equipped with an ESI ion source operating in positive mode. The obtained intact spectra were processed and deconvoluted using UniDec software [30]. Further confirmation of the recombinant sequence was performed by a top-down proteomic sequencing. The sample was desalted, converted into the 100 mM ammonium acetate (Sigma-Aldrich, Saint Louis, MO, USA) using Micro Bio-Spin P6 gel columns and further diluted by 0.5% formic acid (Sigma-Aldrich, Saint Louis, MO, USA) in acetonitrile/H_2_O (1:1, v/v) to an approximate concentration of 300 μg/mL. Intact spectrum was obtained as described above. The most dominant charge states were selected and isolated for fragmentation, and the obtained MS/MS spectra were deconvoluted and exported using Bruker Data Analysis software. The exported list of fragment ions was then loaded into ProSight Lite [31] and compared with the expected sequence of rhGH.

All 34 samples were further quantified by liquid chromatography using two detection methods for comparison: UV spectrometry and low-resolution mass spectrometry. The separation was performed using Agilent 1290 Infinity II LC system with 1100 UV detector, coupled to Agilent 6470 Triple Quadrupole (Agilent Technologies, Santa Clara, CA, USA). The separation was performed using an XBridge Premier Protein BEH C4 column, 100 × 2.1 mm, 2.5 μm (Waters, Milford, MA, USA), and mobile phases consisted of 0.1% formic acid in water (A) and acetonitrile (B). The flow rate was 0.300 mL/min and the gradient was as follows: 0.0–1.0 min 20% B, 1.0–6.0 min raised to 100% B, 6.0–7.5 min kept constant at 100% B, 7.5–9.0 min dropped to 20%. After each run, the column was equilibrated for 1.5 min. For UV detection, chromatograms were acquired at 280 nm. The scan of all MS was performed in the range of m/z 1300–1600, and total ion chromatogram (TIC) peaks were evaluated for quantification. Each sample was diluted 100× by the mobile phase and 1 μL of the solution was injected to the system. The quantification was performed using an external calibration that was prepared by diluting a standard hGH (PeproTech EC, NJ, USA) solution.

## 3. Results and Discussion

Given the intended application of the method—analysis of confiscated nutritional supplements—a competitive ELISA format was considered appropriate. This format not only allows for reliable quantification of full-length hGH molecules but also offers the potential for future extension to the detection of related low-molecular-weight fragments, which are increasingly used as doping agents. In contrast to the sandwich ELISA format, which uses two antibodies binding to distinct epitopes, competitive assays are more suitable for detecting such fragments and chemically modified variants, which typically expose only a single epitope.

### 3.1. Commercial Kit Used for hGH Detection in Nutritional Supplements

To assess the suitability of a commercial competitive ELISA kit (MyBioSource)—originally designed for clinical and biological applications—for detecting hGH in illicit nutritional supplements, Sample 1 (confiscated by the Police of the Czech Republic) and model samples were used. These samples contained not only the hGH standard from the kit but also various external hGH from different producers (Sigma-Aldrich, Proteintech Group and PeproTech EC), as described in Table 1. The procedure for the entire analysis is detailed in Section 2.3. When using the internal hGH standard solutions, a reproducible calibration curve with an absorbance range of 0.08–2.9 was obtained. In model samples prepared using the internal hGH (whether dissolved in the solution provided as part of the kit or in commonly used ELISA buffers), the expected hGH concentrations were precisely determined. However, the immunoanalysis of Sample 1 and samples with hGH from all three external sources revealed false-negative results. In all cases (whether using the internal solvent or commonly used ELISA buffers), no competition was observed.

This indicates that the antibodies included in the commercial kit recognise and interact well with the internal hGH but interact poorly with other hGH molecules tested, while the type of diluent used had no effect on the determination. One possible explanation is that this kit (like all others on the market) is designed for the detection of hGH in biological materials, and thus the standard included in the kit may be the native, rather than the recombinant, hGH molecule. Similarly, during the production of the antibodies incorporated in the kit, native hGH may have been used as the immunogen. As a result, the antibodies generated might not interact with rhGH due to its potentially different conformation.

Since the commercial kit proved ineffective in detecting hGH in a real sample of an illicit nutritional supplement, it was necessary to develop a proprietary ELISA method.

### 3.2. Selection of Immunoreagents Suitable for ELISA

During the development of the ELISA method, the initial step involved selecting suitable immunoreagents, in particular the hGH standard and a specific primary antibody. As shown in Table 2, the suspect samples are highly diverse; therefore, the ELISA should be capable of detecting the broadest possible range of hGH, which, given the cost of the production process, are very likely recombinant. For comparison, three recombinant full-length (22 kDa) hGH standards from various producers were selected. The standards from Sigma-Aldrich and Proteintech Group were expressed in HEK 293 cells, whereas the hGH standard from PeproTech EC was expressed in *Escherichia coli* (Table 1).

When selecting antibodies, their different origins (polyclonal × monoclonal) and the immunogen used were considered. Commercially available antibodies were intentionally chosen due to their accessibility, high production standards and batch-to-batch consistency, which supports the robustness of the assay and facilitates its broader application in routine control laboratories. Most antibody producers list rhGH as an immunogen without specifying the expression system. Three of these antibodies were compared in this study: two monoclonal antibodies from Millipore Corporation and Exbio and one polyclonal antibody from Sigma-Aldrich. A monoclonal antibody directly targeting the epitope between amino acids 151–189, supplied by Santa Cruz Biotechnology, was also included (see Table 3).

**Figure 1:**
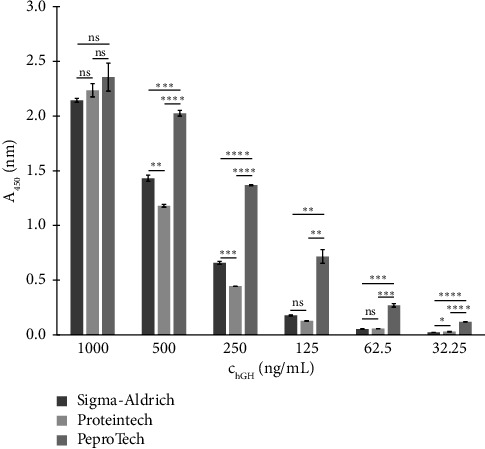
Direct binding of antibody produced by Santa Cruz Biotechnology (concentration of 0.2 μg/mL) to immobilised hGH standards from various producers (Sigma-Aldrich, Proteintech Group and PeproTech EC). Vertical errors bars correspond to the standard deviations of the data points (*n* = 3). One-way ANOVA was used for comparisons, followed by Tukey's multiple comparisons test. Significance levels are indicated as follows: ns = not significant, ^∗^*p* < 0.05, ^∗∗^*p* < 0.01, ^∗∗∗^*p* < 0.001 and ^∗∗∗∗^*p* < 0.0001. Statistical analysis was performed using GraphPad Prism (Version 9.5.1).

Initially, interactions between individual antibodies and all immobilised standards were examined across a broad concentration range using the direct binding method described in Section 2.4.4. For each antibody (listed in Table 3), the intensity of interactions with each tested standard varied considerably. An example of the binding of the antibody from Santa Cruz Biotechnology to all three immobilised standards is shown in Figure 1. The degree of variation was evaluated using a one-way ANOVA test, followed by Tukey's multiple comparisons test, and the significance levels were indicated. This antibody showed the strongest interaction with PeproTech's hGH standard over the wide concentration range tested. A similar trend was also observed for the other antibodies (from Millipore Corporation, Sigma-Aldrich and Exbio), which led to the selection of the PeproTech hGH standard for further experiments.

The results of direct binding of all antibodies to the immobilised PeproTech hGH standard showed that the highest interactions were achieved with antibodies from the following two sources: Santa Cruz Biotechnology and Exbio. Therefore, these antibodies, which in addition were reported by the manufacturers to exhibit high specificity for hGH, were then used to develop an indirect competitive ELISA.

### 3.3. Newly Developed Competitive ELISAs

The assays were designed as indirect competitive ELISAs using monoclonal antibodies targeting the epitope between amino acids 151–189 (antibody from Santa Cruz Biotechnology) or an unspecified epitope of the hGH molecule (antibody from Exbio). The hGH standard was from PeproTech EC. To optimise the ELISA assay, checkerboard titrations were performed to determine appropriate concentrations of all immunoreagents (i.e., immobilised standard and primary and secondary antibody). Our aim was to achieve a maximum absorbance of around 1.0 and the lowest I_50_ values for the calibration curve. Additionally, the appropriate composition of the reaction buffer was selected, and the conditions for each incubation step (time and temperature) were tested to ensure that the resulting methods were as sensitive, cost-effective and simple to perform as possible. The optimised conditions for the hGH immunoassays are summarised in Table 4, and the procedure is described in Section 2.4.5.

**Figure 2:**
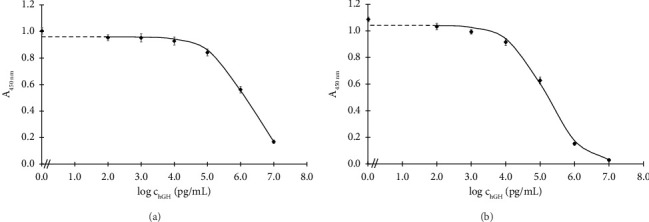
Representative calibration curve for new indirect competitive ELISA with primary Santa Cruz Biotechnology antibody (a) and with primary Exbio antibody (b). Vertical errors bars correspond to the standard deviations of the data points (*n* = 6).

To assess significant analytical parameters of the assembled formats, hGH calibration curves in concentration range of 0.1–10,000 ng/mL were constructed (Figures 2(a) and 2(b)). The assays were carried out under the optimised conditions. Based on the standard curves, LOD, I_50_ and the linear working range were calculated (Section 2.4.6), and they are listed in Table 4.

The Santa Cruz antibody–based assay exhibited an I_50_ of 1466 ng/mL and a LOD of 18.4 ng/mL. Meanwhile, the Exbio antibody–based assay achieved a markedly lower I_50_ of 147 ng/mL and a significantly lower LOD of 0.4 ng/mL.

The Exbio antibody–based competitive assay even exhibits slightly higher sensitivity than the sandwich ELISA developed by Yuki and Kato, which was specifically designed for the 22 kDa hGH variant and had an LOD of 1 ng/mL [32]. The sensitivity of the Exbio antibody–based assay is comparable to that of a sandwich ELISA developed for the quantification of hGH molecules presenting helix 4, with an LOD of 0.12 ng/mL [33], as well as to an LC-MS/MS method targeting the 22 kDa-hGH isoform, which included immunopurification using an anti-GH antibody and had a reported LOD of 0.5 ng/mL [34].

Currently, several types of commercial ELISA kits for hGH detection are available on the market, based on the sandwich format of this method. While the competitive Santa Cruz antibody–based ELISA presented in this study has a higher LOD than all commercial sandwich ELISA kits, the sensitivity of the Exbio antibody–based assay is comparable to some of them, including the sandwich ELISA kit from MyBioSource [35] (LOD 0.5 ng/mL), from Anogen [36] (LOD < 0.5 ng/mL) and from Antibodies [37] (LOD < 0.1 ng/mL). Notably, it is even more sensitive than the LSBio kit [38], which has an LOD of 2.5 ng/mL.

Since the sandwich format is generally significantly more sensitive than the competitive format, several producers have successfully developed kits for hGH detection with LODs in the pg/mL range or even lower. For example, Bio-Techne [39] achieved an LOD of 7.18 pg/mL; Invitrogen [40], Sigma-Aldrich [41] and RayBiotech [42] all report an identical LOD of 4 pg/mL; Antibodies [43] and OriGene [44] also report an identical LOD of < 2 pg/mL; Abcam [45] reports an LOD of 1.6 pg/mL, while Proteintech [46] has achieved an LOD as low as 0.5 pg/mL.

Compared to the only two available competitive ELISA kits, the newly developed competitive ELISA using Exbio antibodies exhibits either comparable sensitivity (e.g., to the LSBio kit [47] with an LOD of 0.39 ng/mL) or higher sensitivity (compared to the MyBioSource kit [48] with an LOD of 1.04 ng/mL).

However, all commercial ELISA methods were designed exclusively for detecting hGH in biological samples, and none have been specifically developed for analysing illicit nutritional supplements. In contrast, both new methods—thanks to the selected antibodies and standard—show promise for this application. Although the Santa Cruz antibody–based assay exhibits higher I_50_ and LOD values compared to the Exbio antibody–based assay, it employs antibody that targets a precisely defined epitope located between amino acids 151 and 189. This epitope is part of the C-terminal region of the hGH molecule, from which a group of synthetically prepared peptides (e.g., AOD9604) has been derived. These peptides are currently used in doping as substitutes for full-length hGH. Owing to the competitive format of the newly developed ELISA method, the Santa Cruz antibody is more likely to serve as an effective tool for detecting all related substances. Consequently, methods based on both antibodies (i.e., not only those from Exbio, which exhibit better analytical parameters, but also those from Santa Cruz Biotechnology) were included in the analysis of suspect nutritional supplement samples.

### 3.4. Detection of hGH Concentration in Black Market Products

**Figure 3:**
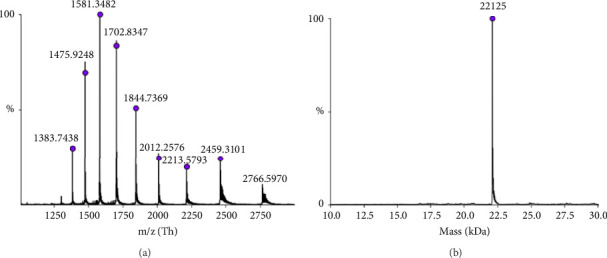
Intact mass spectra of Sample 1. (a) Electrospray mass spectrum with several multiply charged ions, characteristic for different charge states of a single protein. (b) UniDec deconvolution of this spectrum, with molecular mass 22,125 Da confirming the identity of the recombinant hGH.

The identity of hGH in the tested products was confirmed through detailed mass spectrometric analysis of a representative sample (Sample 1), which was desalted and analysed using high-resolution intact mass spectrometry. As shown in Figure 3, the results confirm the accurate molecular mass of rhGH. Additionally, the sequence of hGH was examined using top-down mass spectrometry (Section 2.5), with results summarised in the Supporting file (Figure S1 and Table S1). These findings align well with the known sequence, further verifying the sample's identity.

The newly developed ELISA assays were then employed for the confirmation and quantification of the hGH in 34 suspect black market products. The measured concentrations were compared with product label claims and with quantitative LC data. As shown in Table 5, both specific antibodies (from Santa Cruz Biotechnology and from Exbio) were able to detect the presence of hGH in all analysed samples and were in good agreement with both LC-UV and LC-MS. A comparison of each ELISA assay with LC-MS results was further conducted using Passing–Bablok regression, with detailed methodology available in the Supporting file (section: Methodology of the Passing–Bablok Regression). Both regressions confirmed the validity of a linear model using the CUSUM test. The lower values measured by both ELISA assays compared to LC-MS, as indicated by the Passing–Bablok analysis, could be explained by the potential presence of denatured hGH that, although retaining the same primary sequence and therefore detectable by LC-MS, was not captured by ELISA due to a lack of interaction with the antibody. The ELISA assay using the Exbio antibody showed better agreement with LC-MS and a lower residual SD than the assay based on the Santa Cruz Biotech antibody (Figure S4). Both LC-UV and LC-MS chromatograms, along with calibration curves, are provided in Figures S2 and S3 of the Supporting file.

## 4. Conclusion

An existing commercial competitive ELISA kit, designed for the determination of hGH in biological materials, proved ineffective in detecting hGH in illegal nutritional supplements abused by athletes. Therefore, indirect competitive enzyme-linked immunoassays, specifically designed for this type of samples, were developed. Two primary antibodies, one from Santa Cruz Biotechnology and one from Exbio, were selected based on their performance in the binding studies. The LOD for the immunoassay using the Santa Cruz Biotechnology antibody was 18.4 ng/mL and that for Exbio antibody system was 0.4 ng/mL. Both newly developed methods successfully detected hGH in all 34 suspect products analysed, with the results confirmed by LC-UV and LC-MS. This study represents the first published ELISA methods developed specifically for the analysis of illegal nutritional supplements, which could offer a required verification tool for mass spectrometry currently employed by the Police of the Czech Republic in the analysis of confiscated products. In addition, these ELISA methods, utilising specific antibodies, enable the unambiguous confirmation of hGH identity in suspect samples. Moreover, applying these methods to a larger number of samples may further consolidate their role in routine analysis and enhance their practical applicability.

## Figures and Tables

**Table 1:** hGH standards from several producers.

Name of product	Producer	Product number	Expression organism
HGH human	Sigma-Aldrich (Saint Louis, MO, USA)	SRP6167	HEK 293
Human growth hormone	Proteintech Group, Inc. (Rosemont, IL, USA)	HZ-1007	HEK 293
Human growth hormone	PeproTech EC, Ltd. (Cranbury, NJ, USA)	100–40	*Escherichia coli*

**Table 2:** Black market products confiscated by the Police of the Czech Republic (Samples 1–13) and provided by analytical laboratory Janoshik s.r.o. (Samples 14–34).

Sample number	Product label	Packing	Declared amount of hGH (IU)	Declared amount of hGH (mg)	Expected concentration of hGH in reconstituted samples (mg/mL)
1	HGH	Injection vial	10	—	3.3
2	Somatropo	Injection vial	10	3.7	3.7
3	Somatropin	Injection vial	10	3.33	3.3
4	Sitropin	Injection vial	10	3.3	3.3
5	Nanotrop	Injection vial	5	1.65	3.3
6	Somatex	Injection solution in vial (5 mL)	50	16.65	3.3
7	Somatropin	Injection vial	10	3.7	3.7
8	Without label	Injection vial	—	—	—
9	Without label	Injection vial	—	—	—
10	SOMATEX	Injection solution in vial (5 mL)	50	—	3.3
11	SOMATROP-LAB	Injection solution in vial (5 mL)	50	16.65	3.3
12	Without label	Injection solution in vial	—	—	—
13	SOMATROPIN	Injection solution in vial (5 mL)	50	16.7	3.3
14	Somatropin	Injection vial	10	3.7	3.7
15	BPTROPIN	Injection vial			3.3
16	Without label	Injection vial	—	—	—
17	Without label	Injection vial	—	—	—
18	Somatropin	Injection vial	10	—	3.3
19	Without label	Injection vial	—	—	—
20	Without label	Injection vial	—	—	—
21	HUTROPE LinePen	Injection solution in pen (3 mL)	—	10	3.3
22	SOMATROPIN 191AA	Injection vial	15	—	5.0
23	Chinese written label	Injection vial	—	—	—
24	Without label	Injection vial	—	—	—
25	Without label	Injection vial	—	—	—
26	Without label	Injection vial	—	—	—
27	Without label	Injection vial	—	—	—
28	Dragontropin	Injection vial	10	—	3.3
29	hGH (SynPHARMA)	Injection vial	10	3.3	3.3
30	EUROTROPIN	Injection vial	—	3.33	3.3
31	MEDITROPIN	Injection vial	10	—	3.3
32	Without label	Injection vial	—	—	—
33	SOMATROPIN (SONNAGEN)	Injection vial	12	—	4.0
34	Without label	Injection vial	—	—	—

**Table 3:** Tested antibodies against hGH.

Name of product	Producer	Product number (RRID)	Type	Concentration (mg/mL)	Targeted to
Anti-somatotropin/GH1, clone 1H1.1	EMD Millipore Corporation (Temecula, CA, USA)	MABC531 (−)	Mouse monoclonal	1	Unspecified epitope of hGH
Anti-HGH1 antibody	Sigma-Aldrich (Saint Louis, MO, USA)	HPA042470 (AB_2678013)	Rabbit polyclonal	0.4	Unspecified epitopes of hGH
Anti-somatotropin/GH antibody (E-7)	Santa Cruz Biotechnology, Inc. (Dallas, TX, USA)	sc-374266 (AB_10989917)	Mouse monoclonal	0.2	Epitope between AA 151-189
Anti-hGH antibody clone GH-45	Exbio Praha, a.s. (Czech Republic)	11-126-C100 (AB_10734039)	Mouse monoclonal	0.5	Unspecified epitope of hGH

**Table 4:** Parameters of the assembled indirect competitive hGH ELISAs.

	ELISA with primary antibody	
	Santa Cruz Biotechnology	Exbio
Concentration of the immobilised hGH (ng/mL)	80	100
Concentration of the primary antibody (ng/mL)	50	125
Dilution of the secondary antibody (v/v)	1:3000	1:3500
I_50_ (ng/mL)	1466	147
Linear working range (ng/mL)	260–8290	30–774
LOD (ng/mL)	18.4	0.4
Intra-assay variability—CV (%)	< 8.6	< 9.3
Inter-assay variability—CV (%)	< 12.4	< 11.7

**Table 5:** Comparison of the concentrations of hGH in 34 nutritional supplements, as declared and detected by newly developed competitive ELISA assays, LC-UV and LC-MS.

		Expected concentration of hGH in reconstituted samples (mg/mL)	Detected hGH (mg/mL)			
			ELISA with primary antibody		LC-UV	LC-MS
Sample number	Product label		Santa Cruz Biotechnology	Exbio		
1	HGH	3.3	2.8 ± 0.7	4.0 ± 0.4	2.5 ± 0.5	3.0 ± 0.7
2	Somatropo	3.7	3.3 ± 0.3	3.7 ± 0.5	4.1 ± 0.2	4.8 ± 0.1
3	Somatropin	3.3	3.3 ± 0.6	2.8 ± 0.5	3.3 ± 0.1	3.7 ± 0.1
4	Sitropin	3.3	1.3 ± 0.3	2.3 ± 0.2	2.7 ± 0.3	3.5 ± 0.4
5	Nanotrop	3.3	1.3 ± 0.3	3.3 ± 0.2	3.8 ± 0.2	4.2 ± 0.1
6	Somatex	3.3	1.1 ± 0.4	3.7 ± 0.3	2.9 ± 0.1	3.1 ± 0.0
7	Somatropin	3.7	1.9 ± 0.2	2.8 ± 0.2	3.6 ± 0.4	4.2 ± 0.3
8	Without label	—	1.9 ± 0.3	3.2 ± 0.3	3.1 ± 0.3	3.9 ± 0.1
9	Without label	—	4.5 ± 0.8	4.9 ± 0.4	4.3 ± 0.4	5.3 ± 0.3
10	SOMATEX	3.3	1.9 ± 0.1	2.3 ± 0.2	2.6 ± 0.1	2.1 ± 0.0
11	SOMATROP-LAB	3.3	3.3 ± 0.6	3.9 ± 0.5	3.9 ± 0.0	3.7 ± 0.1
12	Without label	—	4.2 ± 0.5	4.4 ± 0.9	4.3 ± 0.2	3.9 ± 0.1
13	SOMATROPIN	3.3	3.7 ± 0.4	3.8 ± 0.3	3.7 ± 0.2	3.4 ± 0.0
14	Somatropin	3.7	2.2 ± 0.3	3.8 ± 0.5	3.1 ± 0.2	4.0 ± 0.0
15	BPTROPIN	3.3	3.1 ± 0.1	3.3 ± 0.1	3.8 ± 0.2	4.9 ± 0.1
16	Without label	—	2.8 ± 0.7	5.4 ± 0.6	5.2 ± 0.3	6.0 ± 0.4
17	Without label	—	2.2 ± 0.3	4.6 ± 0.2	3.4 ± 0.2	4.5 ± 0.3
18	Somatropin	3.3	2.6 ± 0.1	2.9 ± 0.3	3.5 ± 0.2	4.5 ± 0.2
19	Without label	—	2.0 ± 0.2	4.2 ± 0.6	3.8 ± 0.3	4.7 ± 0.2
20	Without label	—	2.1 ± 0.1	4.4 ± 0.1	3.6 ± 0.2	4.5 ± 0.2
21	HUTROPE LinePen	3.3	2.0 ± 0.1	2.2 ± 0.6	2.7 ± 0.2	2.5 ± 0.1
22	SOMATROPIN 191AA	5.0	3.6 ± 0.3	3.4 ± 0.1	3.2 ± 0.2	3.7 ± 0.3
23	Chinese written label	—	3.9 ± 0.0	4.6 ± 0.2	5.4 ± 0.1	6.2 ± 0.1
24	Without label	—	4.6 ± 0.2	4.0 ± 0.1	4.7 ± 0.2	5.4 ± 0.2
25	Without label	—	6.7 ± 0.6	7.2 ± 0.6	8.0 ± 0.1	8.4 ± 0.1
26	Without label	—	5.3 ± 0.7	5.2 ± 0.8	6.8 ± 0.4	7.3 ± 0.3
27	Without label	—	6.0 ± 0.8	5.6 ± 0.1	8.2 ± 0.1	8.7 ± 0.1
28	Dragontropin	3.3	2.7 ± 0.1	1.6 ± 0.2	2.8 ± 0.5	3.3 ± 0.6
29	hGH (SynPHARMA)	3.3	6.8 ± 0.1	4.6 ± 0.3	4.8 ± 0.1	5.7 ± 0.1
30	EUROTROPIN	3.3	3.6 ± 0.3	4.4 ± 0.3	3.3 ± 0.2	3.8 ± 0.1
31	MEDITROPIN	3.3	3.4 ± 0.4	3.2 ± 0.1	3.1 ± 0.2	3.8 ± 0.1
32	Without label	—	2.6 ± 0.2	4.0 ± 0.5	4.0 ± 0.1	5.0 ± 0.1
33	SOMATROPIN (SONNAGEN)	4.0	4.8 ± 0.6	4.8 ± 0.9	4.3 ± 0.2	4.9 ± 0.2
34	Without label	—	3.1 ± 0.4	2.2 ± 0.2	3.7 ± 0.1	4.6 ± 0.1

## Data Availability

The majority of the data supporting the findings of this study are included in the article. Additional data are available from the corresponding author upon reasonable request.

## Supporting Information

The Supporting Information file includes the following.

Table S1: list of collision-induced dissociation (CID) fragments corresponding to the expected amino acid sequence of hGH.

Figure S1: results of top-down mass spectrometry (MS) analysis of human growth hormone (hGH).

Figure S2: UV chromatogram of a standard hGH solution at a concentration of 100 μg/mL.

Figure S3: calibration curves used for the quantification of hGH.

Figure S4: Passing–Bablok regression and residual plots comparing results obtained by LC-MS and ELISA methods.
