# Supplementary material for: Development of Competitive ELISAs Suitable for Detection of Recombinant Human Growth Hormone in Illegal Nutritional Supplements
Source: J Anal Methods Chem. 2025 Jun 6;2025:3021570. doi: 10.1155/jamc/3021570 (PMC12165756; doi:10.1155/jamc/3021570)
Supplement: Supporting Information — Additional supporting information can be found online in the Supporting Information section. [file 3021570.f1.docx]

**TABLE S1** List of fragments obtained by top-down MS (CID) of hGH sample that match the expected sequence within 25ppm accuracy. They represent 25% of all detected fragments and explain 16% of the proposed sequence. Most importantly, the N-terminus is well covered by sequencing and the starting amino acids confirm the recombinant origin of the protein. No other available hGH sequence matched the obtained top-down results.

| Name | Theoretical Mass | Observed Mass | Mass Difference (Da) | Mass Difference (ppm) |
| --- | --- | --- | --- | --- |
| B2 | 244.12117 | 244.1157235 | -0.00544647 | -22.3105062 |
| B3 | 345.16885 | 345.1622235 | -0.00662647 | -19.1977546 |
| B4 | 458.25291 | 458.2446235 | -0.00828647 | -18.0827371 |
| B8 | 911.52287 | 911.5046235 | -0.01824647 | -20.0175634 |
| B11 | 1286.70228 | 1286.682824 | -0.01945647 | -15.1211878 |
| B23 | 2727.49072 | 2727.441024 | -0.04969647 | -18.2205815 |
| B24 | 2798.52783 | 2798.483524 | -0.04430647 | -15.8320623 |
| B25 | 2945.59624 | 2945.552024 | -0.04421647 | -15.0110413 |
| B26 | 3060.62318 | 3060.576424 | -0.04675647 | -15.27678 |
| B27 | 3161.67086 | 3161.624024 | -0.04683647 | -14.8138339 |
| B28 | 3324.73419 | 3324.685224 | -0.04896647 | -14.7279343 |
| B29 | 3452.79277 | 3452.741424 | -0.05134647 | -14.8709958 |
| B30 | 3581.83536 | 3581.782324 | -0.05303647 | -14.8070644 |
| B31 | 3728.90377 | 3728.848924 | -0.05484647 | -14.7084694 |
| B32 | 3857.94636 | 3857.889524 | -0.05683647 | -14.7323113 |
| B33 | 3986.98895 | 3986.929824 | -0.05912647 | -14.8298547 |
| B34 | 4058.02606 | 4057.965824 | -0.06023647 | -14.8437851 |
| B35 | 4221.08939 | 4221.029224 | -0.06016647 | -14.253777 |
| B36 | 4334.17345 | 4334.109924 | -0.06352647 | -14.6571123 |
| B39 | 4688.36376 | 4688.297924 | -0.06583647 | -14.0425253 |
| B40 | 4816.42234 | 4816.349924 | -0.07241647 | -15.0353233 |
| B41 | 4944.5173 | 4944.447424 | -0.06987647 | -14.1321109 |
| B42 | 5107.58063 | 5107.518824 | -0.06180647 | -12.1009283 |
| B44 | 5341.68107 | 5341.607124 | -0.07394647 | -13.843295 |
| B46 | 5582.82371 | 5582.744324 | -0.07938647 | -14.2197696 |
| B47 | 5696.86664 | 5696.785024 | -0.08161647 | -14.3265539 |
| B190 | 21945.96167 | 21945.58632 | -0.37534647 | -17.1032135 |
|  |  |  |  |  |
| Y17 | 1898.913105 | 1898.890624 | -0.02248147 | -11.8391236 |
| Y20 | 2255.119065 | 2255.084724 | -0.03434147 | -15.2282278 |
| Y22 | 2501.186495 | 2501.147324 | -0.03917147 | -15.661154 |
| Y144 | 16414.17401 | 16413.89512 | -0.27888147 | -16.9902833 |
| Y155 | 17776.8672 | 17776.54312 | -0.32407147 | -18.2299538 |
| Y157 | 18053.01459 | 18052.70382 | -0.31076147 | -17.2138268 |
| Y158 | 18124.0517 | 18123.72912 | -0.32257147 | -17.7979776 |
| Y159 | 18253.09429 | 18252.78542 | -0.30886147 | -16.921047 |
| Y162 | 18658.24788 | 18657.91992 | -0.32795147 | -17.5767558 |
| Y163 | 18786.30646 | 18786.02582 | -0.28063147 | -14.9380863 |
| Y187 | 21652.78774 | 21652.43532 | -0.35241147 | -16.2755702 |
| Y188 | 21765.8718 | 21765.56052 | -0.31127147 | -14.3008959 |
| Y189 | 21866.91948 | 21866.58122 | -0.33825147 | -15.4686382 |
| Y190 | 21963.97224 | 21963.64242 | -0.32981147 | -15.0160209 |


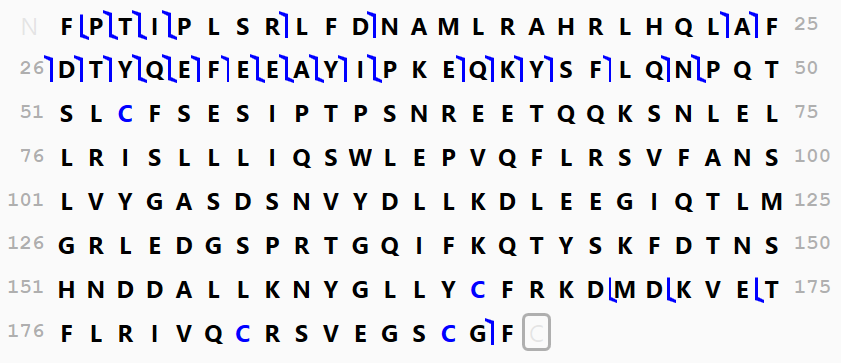


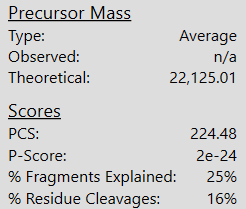


**FIGURE S1** Results of top-down analysis obtained after processing in ProSight Lite.

**B**

**A**


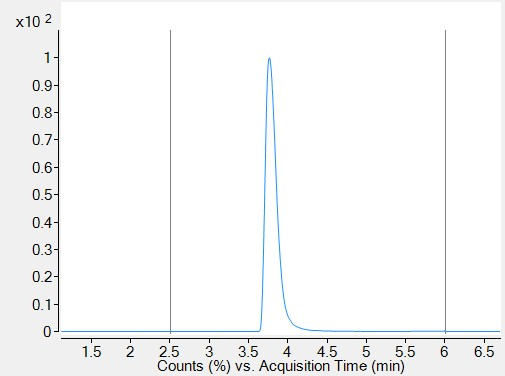

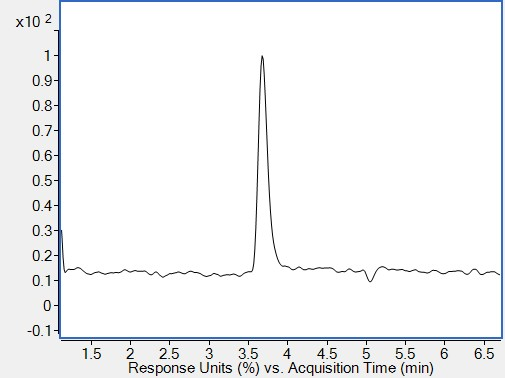


**FIGURE S2**: UV chromatogram at 280 nm (A) and full scan MS chromatogram of *m/z* 1300-1600 (B) of a standard hGH solution (PeproTech EC) at 100 μg/mL


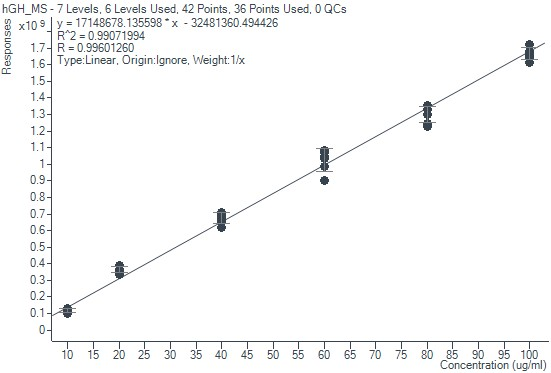

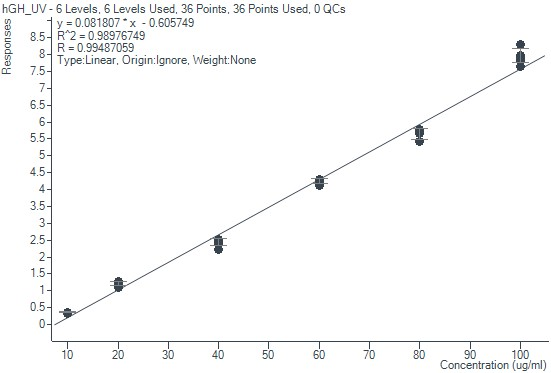


**B**

**A**

**FIGURE S3** Calibration curve used for the quantitative hGH analysis: the peaks from UV chromatograms (A) and MS chromatograms (B) were integrated by the MassHunter software (Agilent Technologies, USA) and the area was plotted against the concentration of the hGH protein in the calibration standard samples.


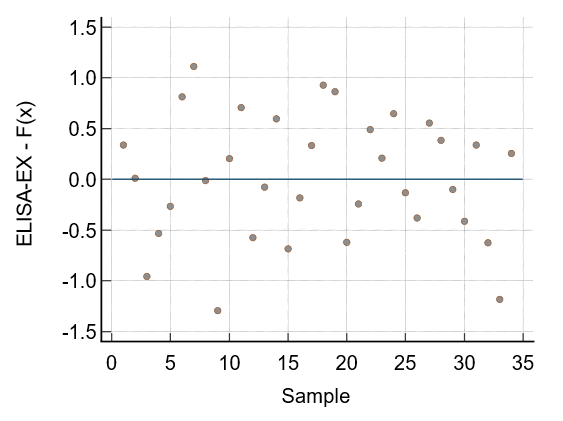

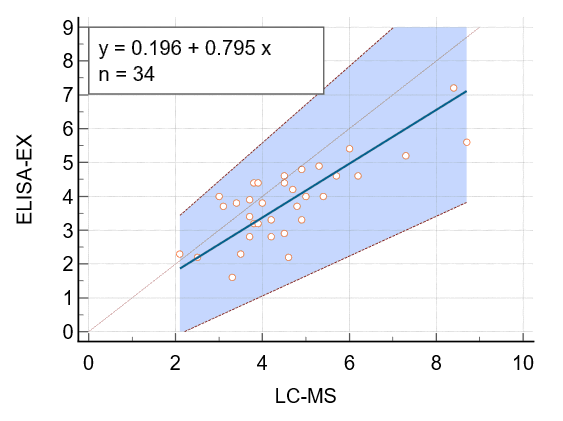
**FIGURE S4** Passing–Bablok regression and residual plots for comparison of ELISA-Exbio antibody with LC-MS; comparison based on the measurement of 34 samples by both methods.

**A**

**B**

A: Passing–Bablok regression for ELISA with antibody from Exbio (ELISA-EX) and mass spectrometry detection (LC-MS). Both coordinates have units of mg/mL. The dotted red line corresponds to y=x (theoretical 45 degrees line).

B: Passing-Bablok regression residuals plot. The plot shows the distance of value obtained by ELISA from the value predicted the by regression for each sample in the dataset. Calculated Residual Standard Deviation was 0.6204, the 1.96 interval that corresponds to 95% level of confidence ranges from -1.2159 to 1.2159.

**Methodology of the Passing – Bablok regression**

The equation y = a + b x defines the Passing-Bablok regression line. If the observations (results) don’t lie on the regression line, then the difference from the expected y-value represents the residuum.

If

1. linear relationship between the two methods being compared is expected
2. normal distribution of residuals is presumed,

then 95% of the residuals should lie in the interval ± 1.96 times the residual standard deviation. This interval defines the random differences between the two methods. Narrower intervals indicate that the methods are in better agreement than methods with wider intervals and *vice versa*.

(reference: Passing H, Bablok W (1983) A new biometrical procedure for testing the equality of measurements from two different analytical methods. Application of linear regression procedures for method comparison studies in Clinical Chemistry, Part I. Journal of Clinical Chemistry & Clinical Biochemistry 21:709-720.)
